# Scaling up testing for COVID-19 in Africa: Responding to the pandemic in ways that strengthen health systems

**DOI:** 10.4102/ajlm.v9i1.1244

**Published:** 2020-05-14

**Authors:** Farouk A. Umaru

**Affiliations:** 1 Department of Global Public Health, United States Pharmacopeia, Rockville, Maryland, United States

In the midst of responding to the coronavirus disease 2019 (COVID-19) pandemic, public health practitioners, agencies and the private sector are partnering to provide urgent emergency solutions to the ongoing crisis. In the words of World Health Organization Director General, Dr Tedros Ghebreyesus, a critical component of this response is to ‘test, test and test’. This need for testing continues to spur multiple innovations in testing techniques, strategies and applications.

As of 08 April 2020, more than 48 different *in vitro* diagnostic devices for COVID-19 diagnosis were listed on the World Health Organization website under the International Medical Devices Regulatory Forum jurisdiction as having received Emergency Use Authorization (EUA) from nine countries, with China authorising 19 devices or technologies (including antibody test kits).^1^ Although no country in Africa has issued an EUA on any of these devices, it is very likely that most of these devices may be marketed or distributed on the continent.

While developed countries like the United States, Italy and Spain have struggled to cope with large-scale testing on multiple devices, many countries in Africa are disproportionately hit by the need for testing because of severe limitations in testing technologies. The lack of Africa-issued EUAs on emerging technologies specific to severe acute respiratory syndrome coronavirus 2 (SARS-CoV-2), the virus responsible for COVID-19, may continue to handicap Africa’s response to the pandemic. But, should African regulatory agencies or the Africa Centres for Disease Control and Prevention (CDC) begin to issue EUAs for emerging technologies, with limited validation information in response to the COVID-19 pandemic?

African Union Member States, through the efforts of Africa CDC and partners, have received technical support to use existing real-time polymerase chain reaction (RT-PCR) instruments to conduct testing, mostly at national reference or equivalent laboratories. Although this technology may be inadequate to entirely meet the scale of testing required for COVID-19 (because of limited numbers of instruments), these instruments are within the existing tiered laboratory network. Leveraging existing RT-PCR instruments for COVID-19 diagnosis is an important step in strengthening health systems on the continent for future emergency pandemics. Responding to the current pandemic in ways that strengthen health systems and that go beyond emergency solutions to consider long-term solutions will benefit the continent as a whole.

The Ebola outbreak in West Africa provides useful lessons on how emergency responses can impact health systems.^2^ During the Ebola outbreak, novel technologies were provided to countries without consideration to the existing tiered laboratory network. As a consequence, some countries have been unable to incorporate those novel technologies into their laboratory networks, which impacts the overall sustainability of their health systems. It is time to remind both national and regional communities on the continent to think beyond the current COVID-19 pandemic so that when Africa emerges on the other side, its health systems will be stronger and more prepared to respond to the next one.

Central questions to keep in mind during the COVID-19 response include: How will countries absorb multiple novel technologies within their health systems post-COVID-19? How will emergency-use-authorised *in vitro* diagnostics be part of national tiered laboratory systems post-pandemic? What role will manufacturers play in initiating long-term evaluation procedures for COVID-19 technologies? Will these technologies be left to countries to manage without adequate support, guidance or capacity? Answers to these questions are critical now.

It is therefore imperative that national regulatory agencies, diagnostics manufacturers and national diagnostics technical working groups not ‘rush’ into issuing or adopting EUAs for new and untested devices outside their networks, but to consider the long-term impact of those technologies on their health systems. Some of these approaches may include:

- Update the current RT-PCR instruments to incorporate COVID-19 testing. As the gold standard for viral testing, countries must work with their existing RT-PCR technology manufacturers to upgrade reagents, kits and software to accommodate COVID-19.^3^ The latest EUA from the United States Food and Drug Administration for the Cepheid *Xpress* cartridge on GeneXpert instruments (Cepheid, Sunnyvale, California, United States) and the Abbott r-SARS-CoV-2 reagents on Abbott m2000 instrument (Abbott Laboratories, Chicago, Illinois, United States) are typical examples.^4^
- National regulatory agencies should develop guidelines that outline clear and unambiguous procedures for issuing EUA for new technologies. These guidelines should incorporate manufacturers’ plans to work with national agencies to incorporate new devices into existing tiered networks as EUAs expire.
- National regulatory agencies should limit EUA approvals to devices that employ the gold standard of RT-PCR in their technologies over antigen-antibody-based, lateral-flow rapid diagnostic test kits, which may not demonstrate comparable sensitivity and specificity to SARS-CoV-2 as with RT-PCR instruments.
- In cases where rapid diagnostic tests are considered (because of urgency to scale up testing), scientifically prudent testing algorithms must be developed by national stakeholders and enforced. In this algorithm, any positive COVID-19 sample from a rapid diagnostic test should be accompanied an RT-PCR-based confirmatory test. In addition, a percentage of negative test samples should also be confirmed with RT-PCR, in order to continuously monitor and confirm the specificity and sensitivity of rapid diagnostic tests.
- National regulatory agencies should seek the support of international technical partners, including the World Health Association, Africa CDC, the African Society for Laboratory Medicine and other non-governmental organisations such as the United States Pharmacopeia and Foundation for Innovative New Diagnostics, to help support and build capacity to rapidly scale up testing for enhanced case management and long-term emergency preparedness.

These strategies and others, supported by national stakeholders, will support African countries in strengthening systems and improve preparedness for emerging pandemics, while building sustainable laboratory systems to help support better healthcare across the continent.

## References

[1] World Health Organization In vitro diagnostics and laboratory technology: Prequalification of IVDs and medical devices. [homepage on the Internet]. Geneva: World Health Organization; c2020 [cited 2020 Apr 8]. Available from: https://www.who.int/diagnostics_laboratory/200408_imdrf_collated_table_8_april_2020.pdf?ua=1

[2] Kieny M-P, Evans DB, Schumets G, et al Health-system resilience: Reflection on the Ebola crisis in western Africa. Geneva: World Health Organization 2014;92:850 10.2471/BLT.14.149278 PMC4264399 25552765

[3] World Health Organization Emergency use listing (EUL), Weekly update. [homepage on the Internet]. date [cited 2020 Apr 4]. Available from: https://www.who.int/diagnostics_laboratory/200403_eul_covid19_ivd_update.pdf?ua=1

[4] United States Food & Drug Administration (FDA) Xpert Xpress SARS-CoV-2 test. [email discussion on the Internet] 2020 March 20 [cited 2020 Apr 8]. Available from: https://www.fda.gov/media/136316/download

